# Genome-Wide Association Study on Male Genital Shape and Size in *Drosophila melanogaster*

**DOI:** 10.1371/journal.pone.0132846

**Published:** 2015-07-16

**Authors:** Baku Takahara, Kazuo H. Takahashi

**Affiliations:** 1 Faculty of Agriculture, Okayama University, Tsushima-naka 1-1-1, Kita-ku, Okayama, 700–8530, Japan; 2 Graduate School of Environmental Science, Okayama University, Tsushima-naka 1-1-1, Kita-ku, Okayama, 700–8530, Japan; Oxford Brookes University, UNITED KINGDOM

## Abstract

Male genital morphology of animals with internal fertilization and promiscuous mating systems have been one of the most diverse and rapidly evolving morphological traits. The male genital morphology in general is known to have low phenotypic and genetic variations, but the genetic basis of the male genital variation remains unclear. *Drosophila melanogaster* and its closely related species are morphologically very similar, but the shapes of the posterior lobe, a cuticular projection on the male genital arch are distinct from each other, representing a model system for studying the genetic basis of male genital morphology. In this study, we used highly inbred whole genome sequenced strains of *D*. *melanogaster* to perform genome wide association analysis on posterior lobe morphology. We quantified the outline shape of posterior lobes with Fourier coefficients obtained from elliptic Fourier analysis and performed principal component analysis, and posterior lobe size. The first and second principal components (PC1 and PC2) explained approximately 88% of the total variation of the posterior lobe shape. We then examined the association between the principal component scores and posterior lobe size and 1902142 single nucleotide polymorphisms (SNPs). As a result, we obtained 15, 14 and 15 SNPs for PC1, PC2 and posterior lobe size with *P*-values smaller than 10^-5^. Based on the location of the SNPs, 13, 13 and six protein coding genes were identified as potential candidates for PC1, PC2 and posterior lobe size, respectively. In addition to the previous findings showing that the intraspecific posterior shape variation are regulated by multiple QTL with strong effects, the present study suggests that the intraspecific variation may be under polygenic regulation with a number of loci with small effects. Further studies are required for investigating whether these candidate genes are responsible for the intraspecific posterior lobe shape variation.

## Introduction

Male genital structures of animals with internal fertilization and promiscuous mating systems have been one of the most diverse and rapidly evolving morphological traits, whereas female genital structures have been relatively invariant during evolution [1]. Although the underlying mechanism of the rapid male genital evolution remains unclear, sexual selection has been regarded as one of the most important factors in male genital evolution [2,3]. Convincing evidence for associations between the male genital morphology and fertilization success have been provided for several species, such as the two water strider species *Gerris lateralis* and *G*. *lacustris* [4,5], dung beetle *Onthophagus taurus* [6], and fly *Dryomyza anilis* [7]. To date, quantitative genetic studies have revealed that the male genital morphology usually has low phenotypic and genetic variations [6,8]; however, the genetic basis of the male genital variation remains unclear.

*Drosophila melanogaster*, one of the best studied model insects, and its closely related species are morphologically very similar; however, the shapes of the posterior lobe, a cuticular projection on the male genital arch are distinct from each other [9]. The posterior lobes, inserted under the female abdominal tergite VII when genital coupling is established, mesh with different parts of the intersegmental membrane between the tergite VIII and the oviscapts [10], suggesting their function to grasp the female oviscape to ensure a stable genital coupling [11]. Masly & Kamimura [12] have revealed that posterior lobes with allospecific features caused more severe damage to females than those with conspecific features, implying a potential coevolution of male and female genital structures through sexual selection. LeVasseur-Viens et al. [13] experimentally demonstrated that the posterior lobe shape did not affect copulation duration or cryptic female choice in *Drosophila* flies, but they also observed that slight artificial alteration to the posterior lobe shape significantly reduced copulatory success, suggesting that the posterior lobe shape is under precopulatory sexual selection. Although the association between the male posterior lobe morphology and mating success is still under investigation, posterior lobes of the *D*. *melanogaster* species group represent a model system for studying reproductive isolation via the lock-and-key mechanism of secondary sexual structures.

To understand the evolutionary divergence of the posterior lobe morphology, it is necessary to characterize both the inter- and intraspecific genetic variation and elucidate the selective forces acting on it [14]. Till date, the genetic basis of the interspecific variation of the posterior lobe morphology has been studied between *D*. *simulans* and *D*. *mauritiana* [15,16] and between *D*. *sechellia* and *D*. *simulans* [17] using a quantitative trait loci (QTL) mapping approach on the interspecific recombinants. Those studies have identified multiple QTL on each chromosome and have revealed that the genetic basis of the interspecific morphological variation in the posterior lobe is predominantly additive with limited epistasis. Among QTL with a large effect in those species, most affect the posterior lobe morphology in the direction of parental trait values. Amongst the several studies on the morphological variation in the posterior lobe, only McNeil et al. [14] described the intraspecific genetic variation for highly inbred *D*. *melanogaster* strains; this study reports three autosomal QTL, contributing to the difference in the posterior lobe shape between two inbred strains of *D*. *melanogaster*. Although a high-resolution fine-mapping QTL approach was adopted, the three QTL encompass 2085 protein coding candidate genes. Higher resolution mapping is necessary for identifying the genes and genetic pathways responsible for the intraspecific variation in the posterior lobe shape.

Mackay et al. [18] have sequenced the whole genome of a panel of inbred *D*. *melanogaster* strains (Drosophila Genetic Reference Panel, DGRP), providing a great opportunity to assess the genetic architecture of quantitative traits at a fine-scale resolution; because DGRP contains a representative sample of naturally segregating genetic variation and has a limited population structure, it is ideal for performing genome-wide association mapping [18]. In the present study, we took advantage of the DGRP strains and the associated single nucleotide polymorphisms (SNPs) with variation in the posterior lobe shape for identifying the candidate genes underlying the intraspecific variation in the posterior lobe shape.

## Materials and Methods

### Flies and experimental conditions

We used DGRP as a source of genetic variation in the male genital shape. DGRP is a collection of *D*. *melanogaster* strains, comprising more than 200 inbred lines derived from the Raleigh, USA population. We randomly selected 155 strains from DGRP and obtained them from the Bloomington Drosophila Stock Center (S1 Table).

To obtain flies for genital morphological measurements, we reared them under a constant condition and tried to minimize environmental variation. Ten females and 10 males aged 5–6 days after eclosion were confined to a plastic vial with standard cornmeal-agar fly medium for 24 h for oviposition at 25°C under a constant light condition in incubators (MIR-254 or MIR-154; SANYO, Osaka, Japan). We then removed the flies and reared the larvae until eclosion. The emerging adult males were collected from each vial and preserved in 70% ethanol; four replicate vials were set up for each line.

### Male genital morphology data acquisition and shape and size analysis

**Fig 1:**
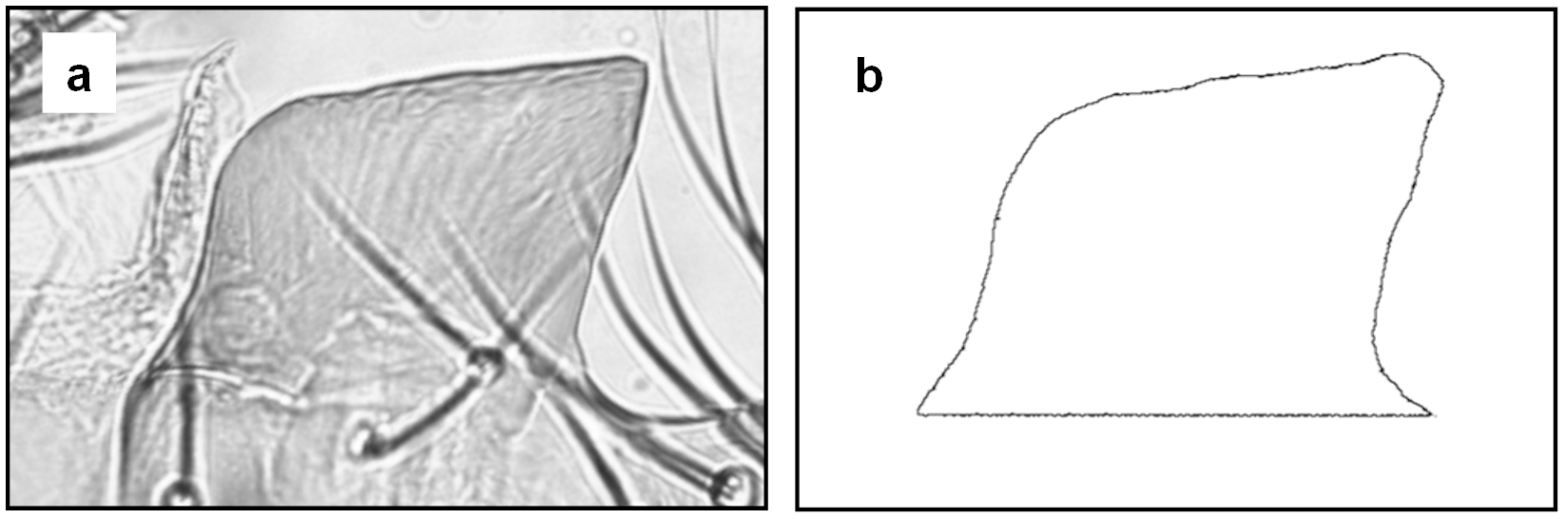
Posterior lobe morphology of *Drosophila melanogaster*. Posterior lobe image (a) and its closed outline (b).

We sampled up to 8 males from each line by pooling the individuals emerged from replicate vials (overall average number of flies sampled per strain was 7.77). The abdomen was dissected from each male and boiled in 0.1 M KOH for 30 min at 60°C to dissolve tissues other than the cuticular exoskeleton. Further, the genital arch on the right side of the body was dissected from each abdomen and mounted on a slide glass in Hoyer’s medium. The posterior lobe was flattened by a coverslip to obtain a two-dimensional shape. We then captured the images of the posterior lobe using an optical microscope (CX41, Olympus, Tokyo, Japan) with a 40× objective lens and CCD camera (WRAYCAM NF500, Wraymer, Osaka, Japan). Each posterior lobe image was manually outlined using Image J [19] (Fig 1). As reported in previous studies on the posterior lobe shape [14,17,20], outlines were closed with an artificial baseline extending from the points where the lateral plate connects to the posterior lobe. To avoid the systemic among-strain variation due to tracing bias in the process of outline acquisition, the same person did all the tracing of the outlines in this study.

Because no reliable homologous landmarks are definable for the posterior lobe, we performed elliptic Fourier analysis (EFA) to describe the outline shape [21]. Series of *x* and *y* coordinates were acquired for each posterior lobe outline and were further translated into a chain code. Before applying EFA, all the outlines were subjected to generalized Procrustes alignment using the artificial baselines for normalizing the size and configuration of the outlines and render the locations of all the posterior lobe outlines comparable. As a result of EFA for each posterior lobe, we obtained 200 Fourier coefficients (50 harmonics of the Fourier series), providing a nearly perfect reconstruction of the original posterior lobe outline. Because we performed generalized Procrustes alignment prior to EFA, we did not employ the Fourier coefficient normalizing procedures suggested by Kuhl & Giardina [21].

The 200 Fourier coefficients were treated as variables describing the shape of the posterior lobe and were analyzed by principal component analysis (PCA). Among the 155 DGRP strains used in the present study, the first principal component (PC1) explained approximately 78% of the total variation while the second principal component (PC2) explained approximately 10% in the 200 Fourier coefficients. Based on the explanatory power distribution among the principal components, we used PC1 and PC2 as the two major shape descriptor variables for further analyses. All shape analyses were performed using the Momocs package [22], and PCA was performed using princomp function in R.

In the current analysis, we used 200 Fourier coefficients (50 harmonics of the Fourier series) to obtain PC1 and PC2 scores, but high number of harmonics in EFA can capture small asperity of the outline and that may result in larger measurement error. To examine this possibility, we obtained outlines of 104 flies from 13 DGRP strains twice, and calculated PC1 and PC2 scores using two to 50 harmonics. We evaluated the measurement error in the PC scores using a repeatability index (*R*) [23]. Based on the repeated measurements, the *R* determines the proportion of variance due to variation between individuals where *R* = 0 indicates that all variance is attributable to variance within individuals (100% measurement error) whilst *R* = 1 indicates all variance is found between individuals (0% measurement error). As a result, both PC1 and PC2 scores showed consistently high repeatability regardless of the number of harmonics (S1 Fig), indicating that the measurement error did not increase with the number of harmonics at least within the range between two to 50 harmonics.

To evaluate posterior lobe size, we used the number of pixels encompassed by the outlines obtained as described above.

### Effects of Wolbachia infection

Approximately half of the DGRP strains are infected with *Wolbachia pipentis*, which is a maternally inherited bacterium [18]; the infection status is publicly available (http://dgrp2.gnets.ncsu.edu/). Because infection of *Wolbachia pipensis* is known to affect various fitness traits in *D*. *melanogaster* [24], we tested the effect of *Wolbachia* infection on genital morphology. To assess whether the *Wolbachia* infection status influenced the male genital morphology, we used the following ANOVA model:

Y= μ+I+S(I)+ ε

where *I* is the infection status (fixed effect), *S* is the DGRP strain (random effect), and *ε* is the error variance.

### Quantitative genetic analysis

To evaluate the broad-sense heritability of the posterior lobe shape quantified with the PC1 and PC2 scores and posterior lobe size, we calculated intraclass correlation coefficients (ICCs) [25,26] by partitioning the total variation into between-genotype and within-genotype components with one-way ANOVA as follows: ICC = (MS_between_ − MS_within_)/(MS_total_), where MS_between_ is the mean square of the between-genotype component, MS_within_ is the mean square of the within-genotype component, and MS_total_ is the sum of these mean squares.

### Genotype–phenotype associations

Because the DGRP strains are a collection of inbred lines derived from a wild population of *D*. *melanogaster*, they have a limited population structure and overall low linkage disequilibrium (LD) [18], which are ideal characteristics for conducting genotype–phenotype association analysis. We associated the mean PC scores and posterior lobe size of each DGRP strain with 1902141 SNPs with minor allele frequency of ≥0.05 in the 155 DGRP strains. Genotype–phenotype associations were analyzed using the ANOVA model:

Y = μ+SNP+ ε

where *SNP* is the SNP genotype effect. We defined an arbitrary threshold *P*-value as 10^-5^, and the SNPs with lower *P*-values were treated as SNPs with a significant effect. Because of the LD effect, individual SNPs are not completely independent and it is difficult to apply adjustment for multiple test properly. In the current study, for descriptive purposes, we assumed independence between SNPs and applied the Benjamini–Hochberg procedure to control the false discovery rate (FDR) [27] and calculated *Q*-values (S1–S3 Tables). We then calculated effect size (Cohen’s *d*) for each candidate SNP to draw more robust conclusion than interpreting the results only using *P*-values. The allelic correlation between those candidate SNPs was quantified with *r* ^2^. We considered protein coding genes located within 1 kb of the significant SNPs as the candidate genes. In this study, the genotype–phenotype association was analyzed using the GWAS analysis pipeline [18].

## Results

### Variation in posterior lobe morphology among DGRP strains

**Fig 2:**
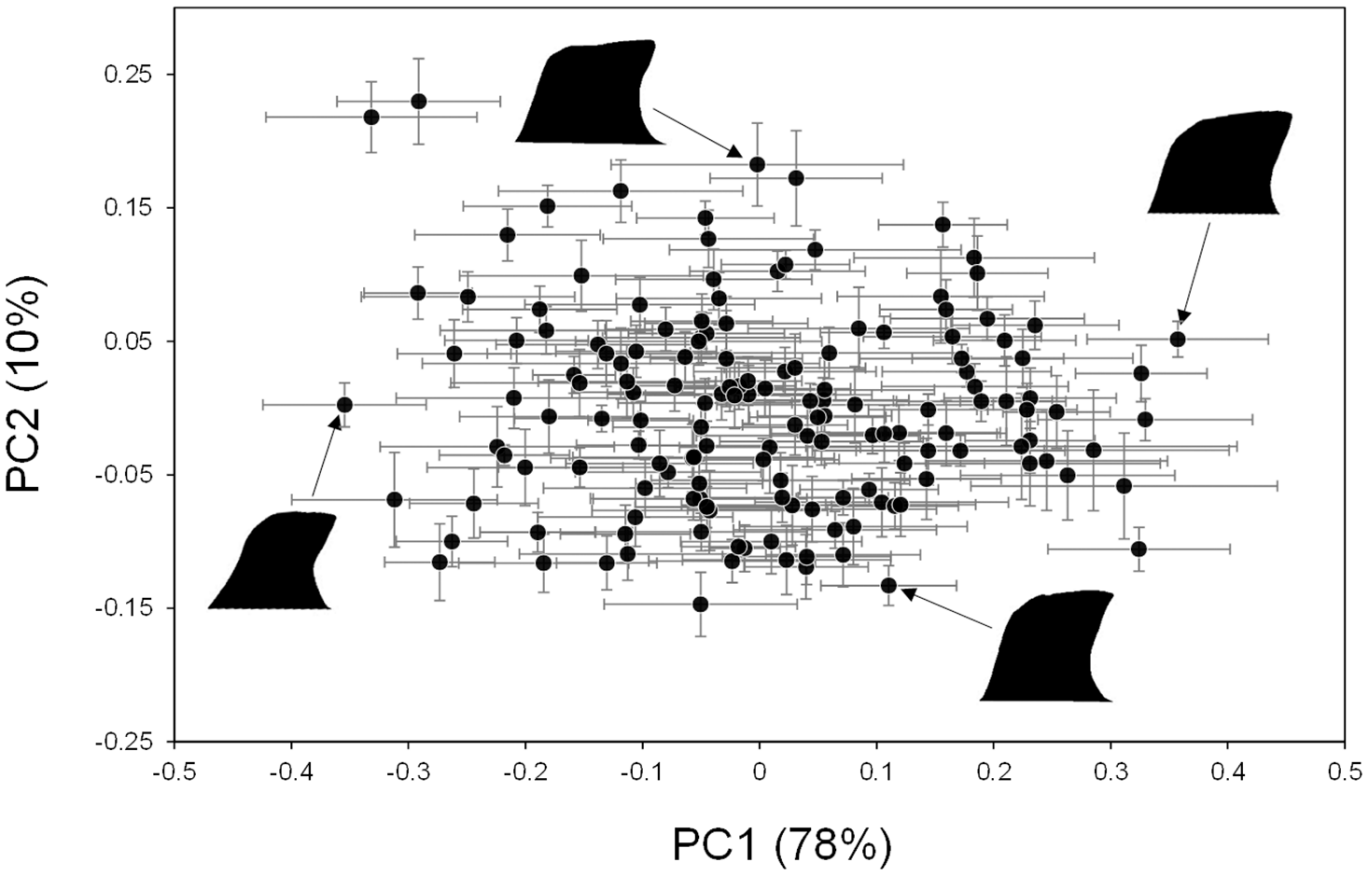
Variation in posterior lobe shape among 155 DGRP strains. Each point represents the average score of each DGRP strain. Error bars represent standard errors. Outlines of posterior lobes show representative examples of the distribution in shape along the two principal component axes (PC1 and PC2).

We found widespread PC scores among the DGRP strains (Fig 2), indicating sufficient intraspecific shape variation, where PC1 describes the degree of beak-like protrusion at the tip of the posterior lobes and PC2 describes its width. The effects of *Wolbachia* infection on PC1 and PC2 scores were not statistically significant (PC1: *P* = 0.304, PC2: *P* = 0.648, posterior lobe size: *P* = 0.876). The broad-sense heritability estimates for PC1, PC2 and posterior lobe size were 0.247, 0.563 and 0.615, respectively, showing moderate to high genetic variation.

### Genome-wide association analyses

**Fig 3:**
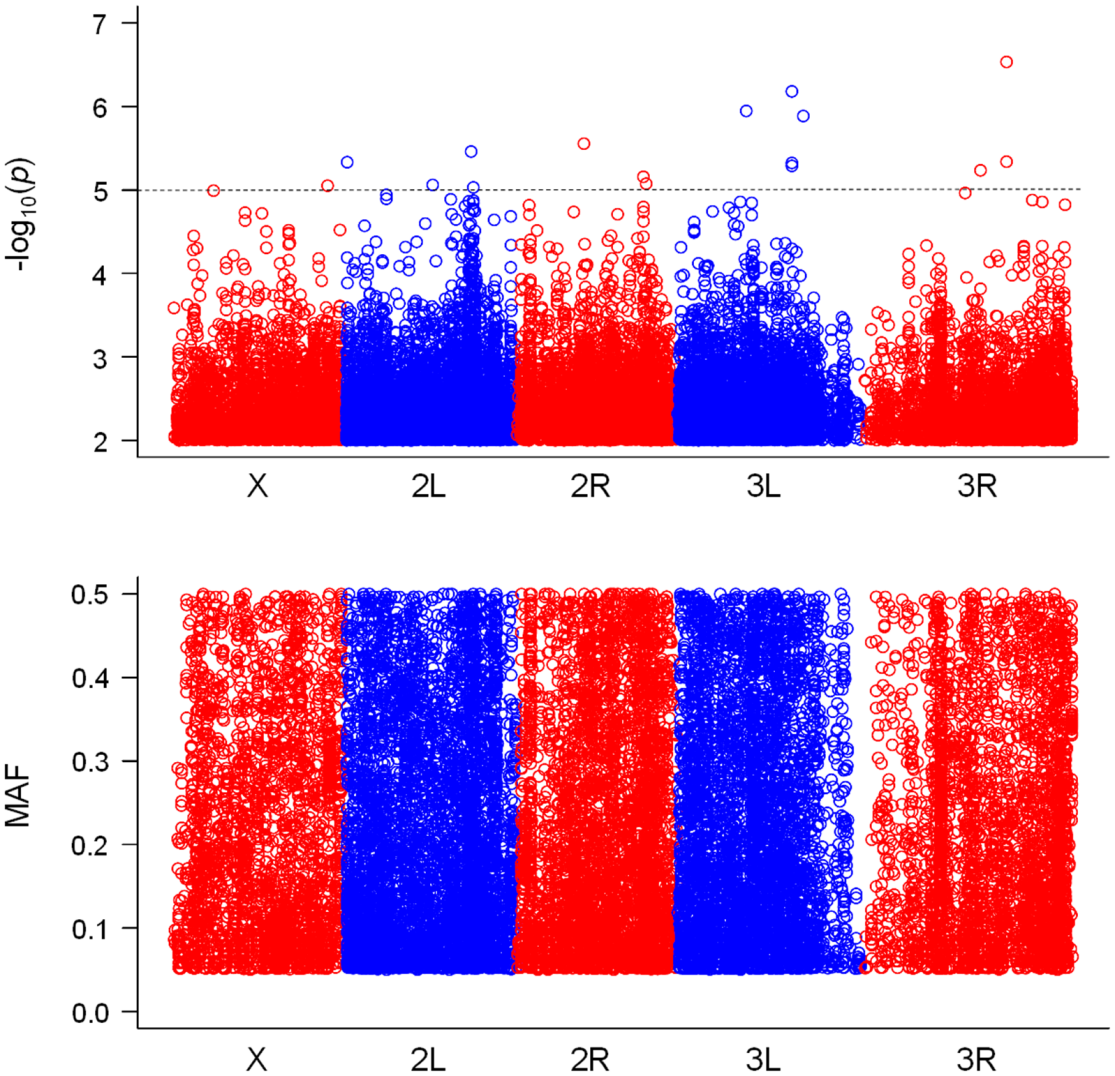
Genome-wide associations for posterior lobe shape characterized with PC1. The top panel shows SNPs with *P*-values smaller than 10^-3^ plotted as -log_10_ (*P*-value), and the bottom panel shows the minor allele frequency (MAF) for each SNP.

**Fig 4:**
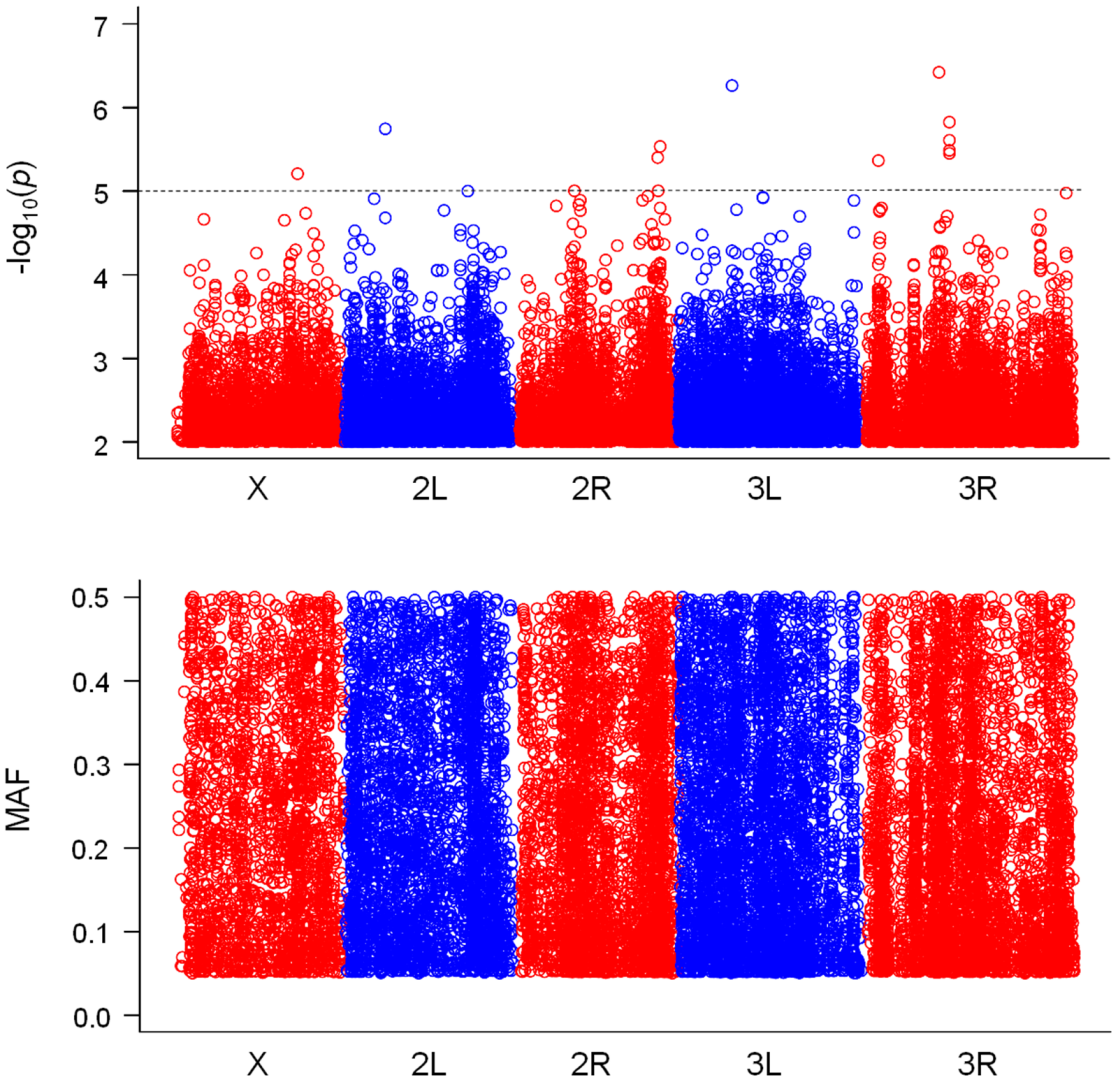
Genome-wide associations for posterior lobe shape characterized with PC2. The top panel shows SNPs with *P*-values smaller than 10^-3^ plotted as -log_10_ (*P*-value), and the bottom panel shows the minor allele frequency (MAF) for each SNP.

**Fig 5:**
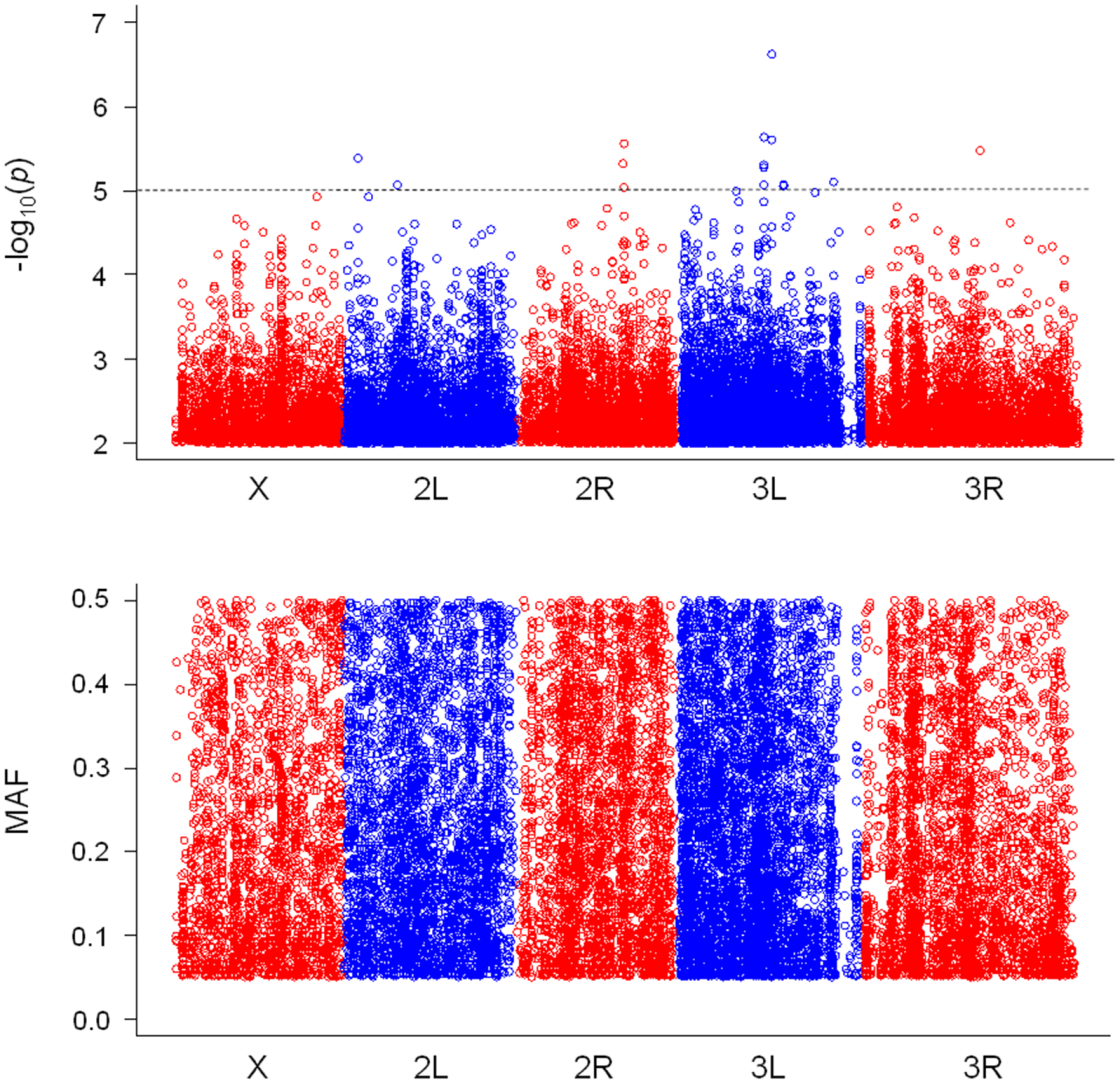
Genome-wide associations for posterior lobe shape characterized with posterior lobe size. The top panel shows SNPs with *P*-values smaller than 10^-3^ plotted as -log_10_ (*P*-value), and the bottom panel shows the minor allele frequency (MAF) for each SNP.

**Fig 6:**
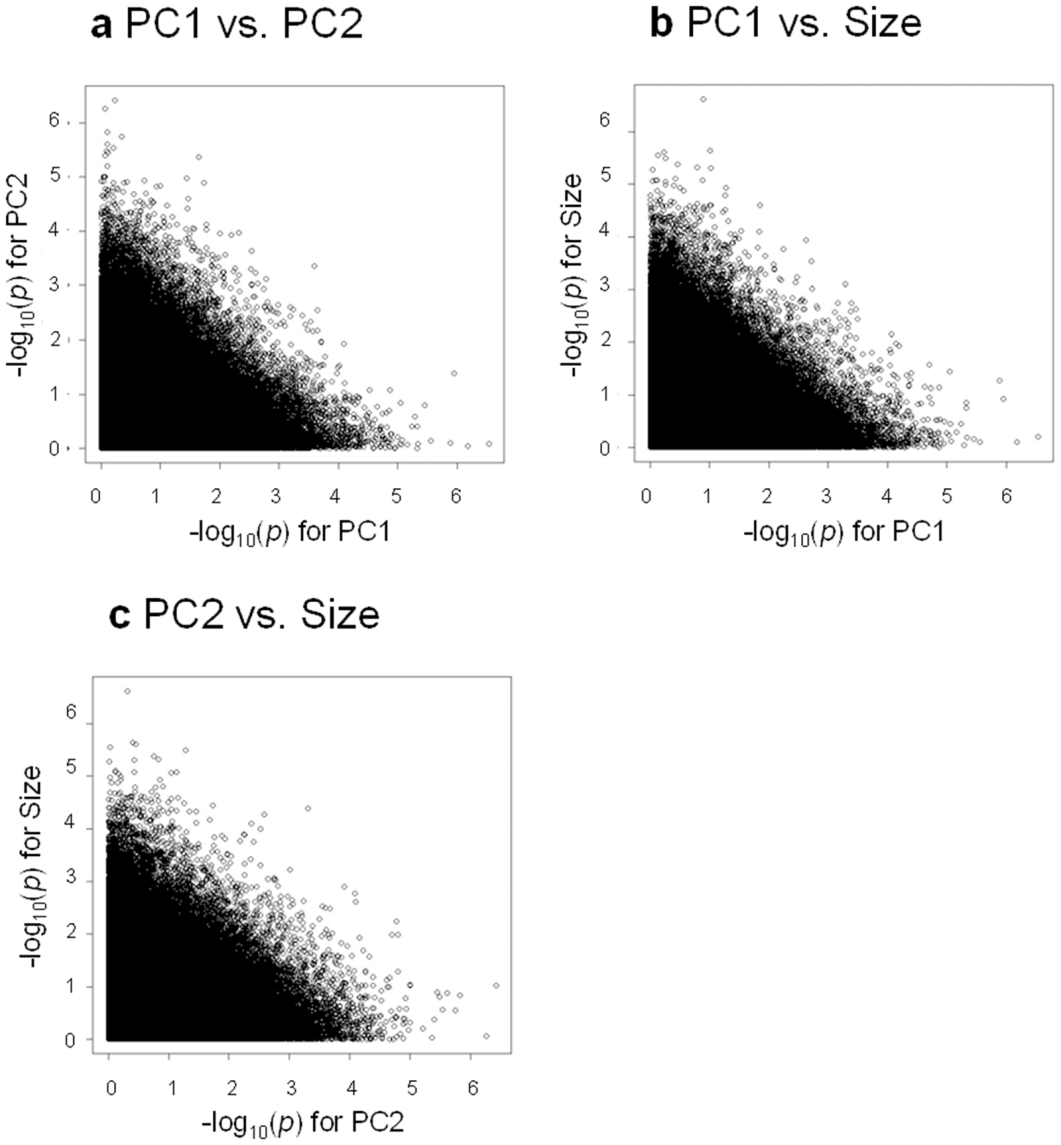
Relationship between the degrees of statistical significance of SNPs for different traits. –log_10_(*p*) for PC1 and PC2 (a), PC1 and posterior lobe size (b), and PC2 and posterior lobe size (c).

**Fig 7:**
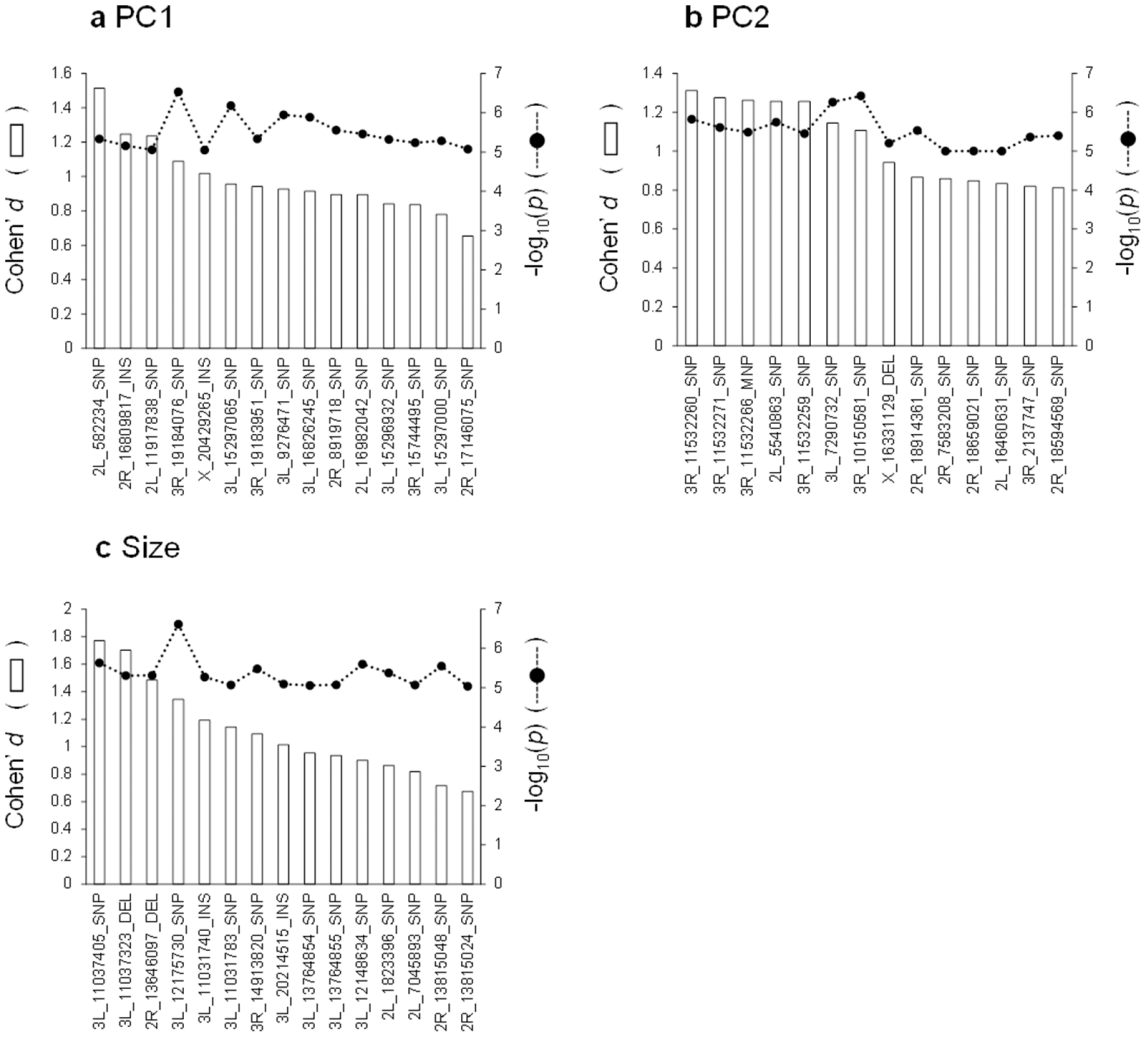
Effect size and statistical significance of candidate SNPs. Cohen’s *d* and -log_10_(*p*) are used as indices of effect size and statistical significance for PC1 (a), PC2 (b) and posterior lobe size (c).

As a result of genotype–phenotype association analyses, we found 15 SNPs for PC1, 14 SNPs for PC2 and 15 SNPs for posterior lobe size with *P*-values smaller than 10^-5^ (Figs 3, 4 & 5, S2, S3 & S4 Tables). According to the quantile–quantile plots of the *P*-values, the observed *P*-values matched well to the null hypothesis except for the SNPs with very small *P*-values, indicating that there were no strong confounders, such as population stratification, in our data (S2 Fig). According to the LD maps for the candidate SNPs for PC1, PC2 and posterior lobe size, LD effect was generally limited to only for a few neighboring SNPs (S3, S4 & S5 Figs). Degrees of statistical significance of SNPs associated with PC1, PC2 and posterior lobe size did not correlate with each other at all (Fig 6), and no SNP had *P*-value smaller than 10^-5^ for two or more traits. Effect size of candidate SNPs showed ranged from 0.65 to 1.51 for PC1, from 0.81 to 1.31 for PC2, and 0.68 to 1.77 for posterior lobe size (Fig 7), indicating that a few SNPs had stronger effect even among the candidate SNPs. Among the 44 SNPs with significant effects, 32 SNPs were located within 1 kb of the protein-coding genes and were considered as candidate genes. In this study, 13, 13 and six protein coding genes were identified as potential candidates for PC1, PC2 and posterior lobe size, respectively (Table 1). McNeil et al. [14] identified three quantitative trait loci (Q1, Q2, and Q3) that influenced the posterior lobe shape of *D*. *melanogaster* on the 2^nd^ and 3^rd^ chromosomes using a fine QTL mapping approach. Seven of the candidate genes identified in the present study were located in 2 of those QTL regions; *GhuRIB* for PC1 was located within Q2, *Invadolysin* and *CG34303* for PC2 were located within Q3, and *CG43693*, *Nrx-IV*, *Rh7* and *bru-3* were located with Q2 (Table 1).

**Table 1:** Candidate genes from this study for PC1, PC2 and posterior lobe size, candidate SNPs associated with them and QTL identified in McNeil et al. (2011).

Trait	Chromosome	Candidate gene	McNeil et al. (2011) QTL
PC1	2L	*Pde1c*	
		*beat-IIIb*	
		*CG44406*	
	2R	*CG8248*	
		*Spt*	
		*CR44465*	
		*snoRNA*:*U3*:*54Ab*	
	3L	*GluRIB*	Q2
		*CG13024*	
	3R	*CG4520*	
		*CG31475*	
	X	*CG11710*	
		*mal*	
PC2	2L	*Hel25E*	
	2R	*blow*	
		*GEFmeso*	
		*CG42306*	
		*GDI interacting protein 3*	
		*SP2637*	
		*sano*	
	3L	*unc-13-4A*	
	3R	*Invadolysin*	Q3
		*CG34303*	Q3
	X	*CG9919*	
		*CG9921*	

## Discussion

In the present study, the intraspecific morphological variation of the posterior lobes of *D*. *melanogaster* was characterized with two major axes, PC1, PC2 and posterior lobe size, representing the degree of beak-like protrusion at the tip of the posterior lobes and its width. Although the intraspecific variation of the posterior lobes we observed was much smaller than the interspecific variation among *Drosophila* species belonging to the *melanogaster* clade [11], it was equivalent or even greater than that previously reported by McNeil et al. [14]. The statistically significant among-strain variation in PC1, PC2 and posterior lobe size and their broad-sense heritability estimates revealed moderate to high genetic variation among the DGRP strains. Because the DGRP strains were established from a single local population (Raleigh, USA), the genetic variation in the posterior lobe shape among the DGRP strains indicates sufficient genetic variation in the intraspecific posterior lobe shape even in a single wild population of *D*. *melanogaster*.

As a result of genome-wide association analyses, we found 32 SNPs associated with PC1 or PC2 scores or posterior lobe size. Those SNPs were distributed to all the major chromosomes of *D*. *melanogaster*, whereas in the study by McNeil et al. [14], the three QTL for posterior lobe shape were found only on the left arms of the 2^nd^ and 3^rd^ chromosomes and the centromere region of the 3rd chromosome. Twelve of these SNPs with significant association did not have any protein coding genes within 1 kb downstream or upstream of their location. Some of those intergenic SNPs (*2L_582234_SNP* for PC1, *3R_11532260_SNP*, 3*R_11532271_SNP* and *3R_11532266_MNP* for PC2) showed relatively high effect size although they did not show strong LD with other candidate SNPs in association with protein coding genes. Those intergenic SNPs may correspond to a genetic variation in the non-coding RNA [28] or cis-regulatory elements of distantly located genes; however, in the present study, we did not follow-up their characterization. The 32 SNPs with significant association corresponded to 33 protein-coding candidate genes in total. Some of the candidate genes identified in this study corresponded to previously identified QTL or genes that were suggested to be involved in genital morphogenesis. Seven of those genes (*GluRIB*, *Invadolysin*, *CG34303*, *CG43693*, *Nrx-IV*, *Rh7* and *bru-3*) were located within the two QTL for the posterior lobe shape of *D*. *melanogaster* identified by McNeil et al. [14]. Two of the candidate genes (*sano*, *CG13024*) were identified to express differentially between sexes in genital imaginal disc in *D*. *melanogaster* [29]. Three of the candidate genes (*mal*, *Hel25E* and *blow*) were identified to express differentially between male genital discs of two *Drosophila* species with morphologically very different posterior lobe shape (*D*. *mauritiana* and *D*. *sechellia*) [20]. Although these twelve genes are good candidate genes responsible for the intraspecific posterior lobe shape variation, their association with posterior lobe shape needs to be examined individually in the future study. Among the candidate genes, *Pde1c* was associated with the SNP with third largest effect size for PC1, and was the only candidate gene that has been previously associated with fertility and male mating behavior in *D*. *melanogaster* [30]. *Pde1c* is one of the six genes that code for cyclic nucleotide phosphodiesterases (PDEs) [31,32]. Because the females found *Pde1c* mutant males as unacceptable and tried to reject them, *Pde1c* mutant males revealed a reduced copulation rate and longer copulation latency [30]. The neuronal expression of *Pde1c* is required for normal copulation behavior, while its non-neuronal expression was suggested to be necessary for fertility [30]. To the best of our knowledge, presently, there is no evidence for the role of *Pde1c* in the morphogenesis of the posterior lobe; however, because *Pde1c* is primarily expressed in the olfactory epithelium [33], it can affect the olfactory perception of larvae and their foraging behavior. Because nutritional condition during the larval period is known to affect the posterior lobe size of *D*. *melanogaster* [34] and could potentially affect posterior lobe shape, *Pde1c* may affect the posterior lobe shape by modifying their foraging behavior. Except *Pde1c*, none of the candidate genes were previously suggested to be associated with posterior lobe morphogenesis or copulation behavior. Further studies are required for investigating whether these candidate genes are responsible for the intraspecific posterior lobe shape variation.

The present study set the threshold *P*-value as 10^-5^ and identified 32 SNPs with significant association, but there were many more SNPs with slightly higher *P*-values (e.g., 223 SNPs for PC1 and 183 SNPs for PC2 with *P*-values smaller than 10^-4^), suggesting that there are many SNPs with small effects on the posterior lobe shape. Previous studies on the intra- and interspecific posterior lobe shape variation were based mainly on QTL mapping approaches and identified multiple QTL with strong effects: three autosomal QTL for intraspecific variation in *D*. *melanogaster* [14]; 15 QTL and 19 QTL for interspecific variation in *D*. *simulans* and *D*. *mauritiana*, respectively [15,35]; and 20 QTL in total for the interspecific variation between *D*. *simulans* and *D*. *sechellia* [17]. Furthermore, Tanaka et al. [16] reported that even within such QTL there are many distinct loci and genes contributing to the posterior lobe shape difference between *D*. *simulans* and *D*. *mauritiana*. In addition to the previous findings showing that the intra- and interspecific posterior shape variation are regulated by a multiple QTL with strong effects, the present study suggests that the intraspecific variation is under polygenic regulation with a number of loci with small effects.

In the current study, we did not correct for multiple tests and use adjusted *P*-value to evaluate the significance of SNPs. It was partly because of the non-independence of the linked SNPs. As Mackay et al. [18] described, linkage disequilibrium (LD) decays to *r* ^2^ = 0.2 on average within 10 base pairs on autosomes and 30 base pairs on the *X* chromosome in DGRP strains, suggesting that there was relatively weak LD structure in our data. If we apply Benjamini–Hochberg procedure under the assumption of independence of the SNPs, no SNPs analyzed showed smaller *Q*-values than 0.05, suggesting that there may be false positive SNPs in our candidate SNPs. The effects of the candidate SNPs and genes need to be examined using mutants or RNAi screening in future studies.

In future studies, a genome-wide association approach should be applied to the intra- and interspecific variation in the posterior lobe shape variation to reveal their underlying genetic architecture at a higher resolution.

## Supporting Information

S1 Fig The relationship between repeatability scores for PC1 (a) and PC2 (b) and the number of harmonics of the Fourier series used for principal component analysis. (TIF)

S2 Fig Quantile–Quantile plots for PC1 (a) and PC2 (b). (TIF)

S3 Fig Linkage disequilibrium (LD) map for the candidate SNPs for PC1. (TIF)

S4 Fig Linkage disequilibrium (LD) map for the candidate SNPs for PC2. (TIF)

S5 Fig Linkage disequilibrium (LD) map for the candidate SNPs for posterior lobe size. (TIF)

S1 Table DGRP strains used in this study. (XLSX)

S2 Table Genome-wide association results for PC1. For SNPs with *P*-values > 10^-3^, ID, minor and major alleles, minor allele frequency (MAF), minor allele count, major allele count, *P*-value and *Q*-value are listed.
(XLSX)

S3 Table Genome-wide association results for PC2. For SNPs with *P*-values > 10^-3^, ID, minor and major alleles, minor allele frequency (MAF), minor allele count, major allele count, *P*-value and *Q*-value are listed.
(XLSX)

S4 Table Genome-wide association results for posterior lobe size. For SNPs with *P*-values > 10^-3^, ID, minor and major alleles, minor allele frequency (MAF), minor allele count, major allele count, *P*-value and *Q*-value are listed.
(XLSX)
