# Correction: Genome-Wide Association Study on Male Genital Shape and Size in *Drosophila melanogaster*

**DOI:** 10.1371/journal.pone.0205301

**Published:** 2018-10-02

**Authors:** Baku Takahara, Kazuo H. Takahashi

There are errors in Table 1. The SNP coordinates used in the genome-wide association analysis are based on Release 5 of the *D*. *melanogaster* reference genome. However, the candidate gene search was performed on the FlyBase in which the coordinates of the genes are based on Release 6 of the *D*. *melanogaster* reference genome. Because the genome sequences used for each release are different, the SNP coordinates needed to be converted to the coordinates of the Release 6 reference genome. Please see the corrected Table 1 here.

**Table 1:** Candidate genes from this study for PC1 and PC2 and QTL identified in McNeil et al. (2011).

Trait	Chromosome	Candidate gene	McNeil et al. (2011)
			QTL
PC1	2L	*Pde1c*	
		*beat-IIIb*	
		*CG44406*	
		*CR44408*	
	2R	*Rx*	
		*Rgk3*	
	3L	*CG7255*	
		*GluRIB*	Q2
	3R	*CG4770*	
		*Sec13*	
		*RpS3*	
	X	*RunxB*	
PC2	2L	*Hel25E*	
	2R	*Obp58d*	
		*Obp58c*	
		*CR43735*	
		*CD30275*	
		*CG42260*	
		*twi*	
		*CG42741*	
		*CG13197*	
	3L	*unc-13-4A*	
	3R	*CG5404*	
		*Osi17*	Q3
	X	*hang*	
Size	2L	*CG31933*	
		*CG31664*	
	2R	*HLH54F*	
	3L	*CG43693*	Q2
		*Nrx-IV*	Q2
		*CAH2*	Q2
		*bru-3*	
		*CG17145*	Q3
		*cdi*	
