# Supplementary figures and images for: Genome-Wide Association Study on Male Genital Shape and Size in Drosophila melanogaster
Source: PLoS One. 2015 Jul 16;10(7):e0132846. doi: 10.1371/journal.pone.0132846 (PMC4504508; doi:10.1371/journal.pone.0132846)

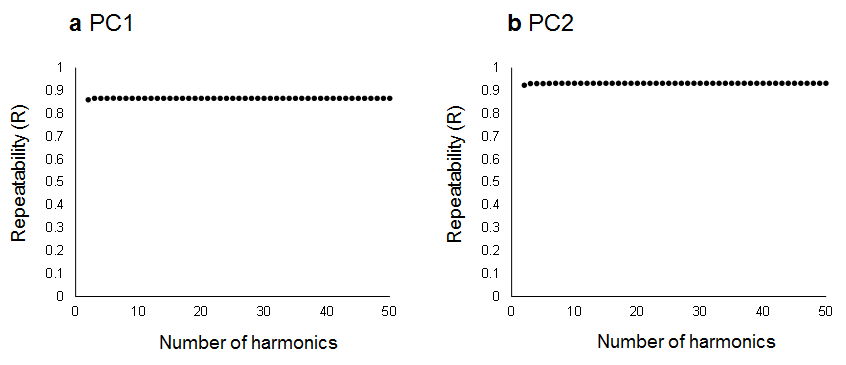

Supplement: S1 Fig — (TIF) [file pone.0132846.s001.tif]

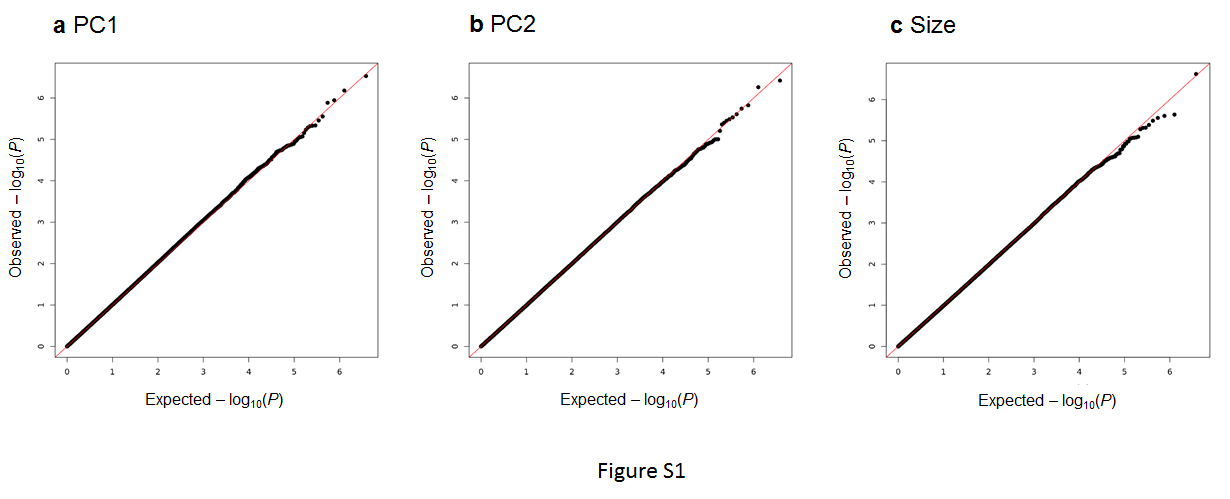

Supplement: S2 Fig — (TIF) [file pone.0132846.s002.tif]

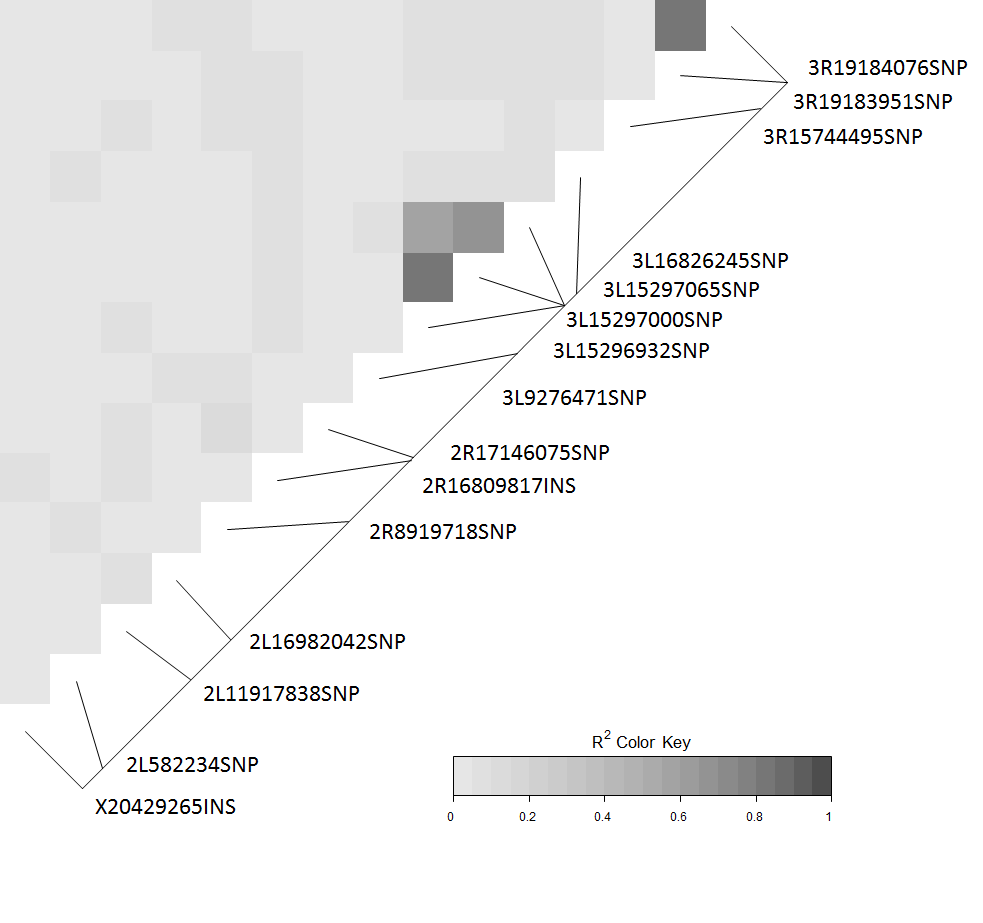

Supplement: S3 Fig — (TIF) [file pone.0132846.s003.tif]

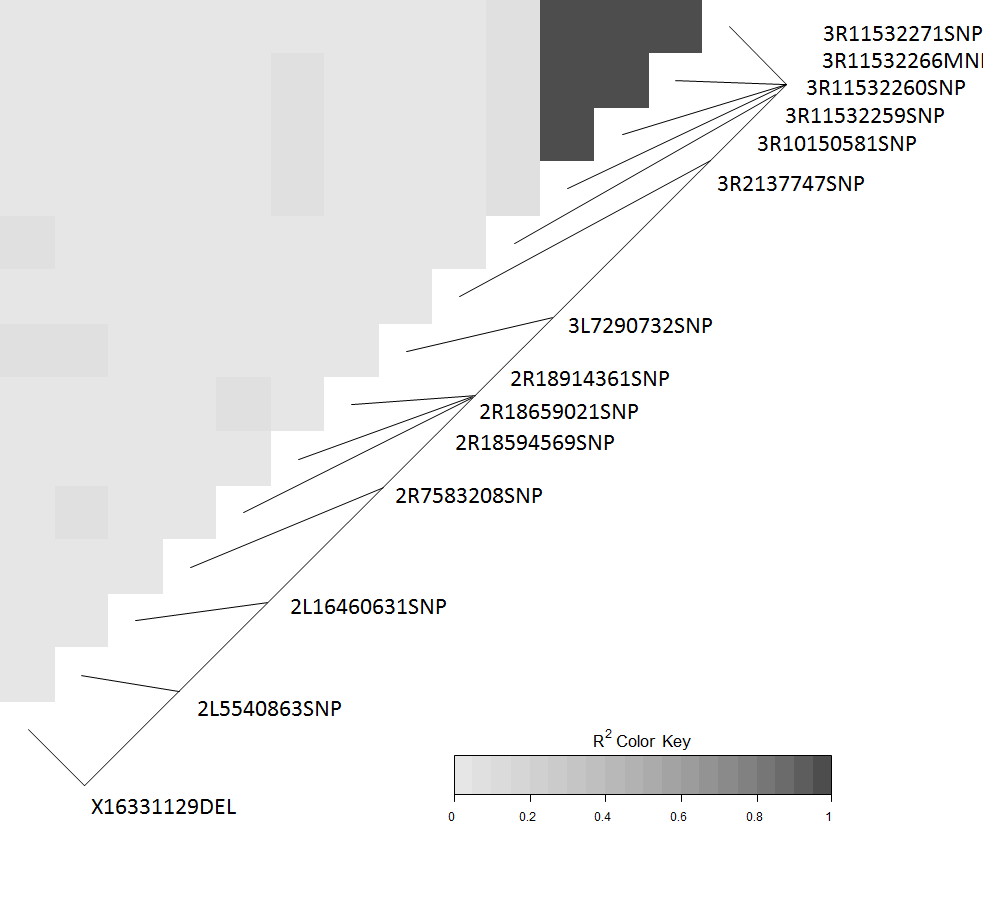

Supplement: S4 Fig — (TIF) [file pone.0132846.s004.tif]

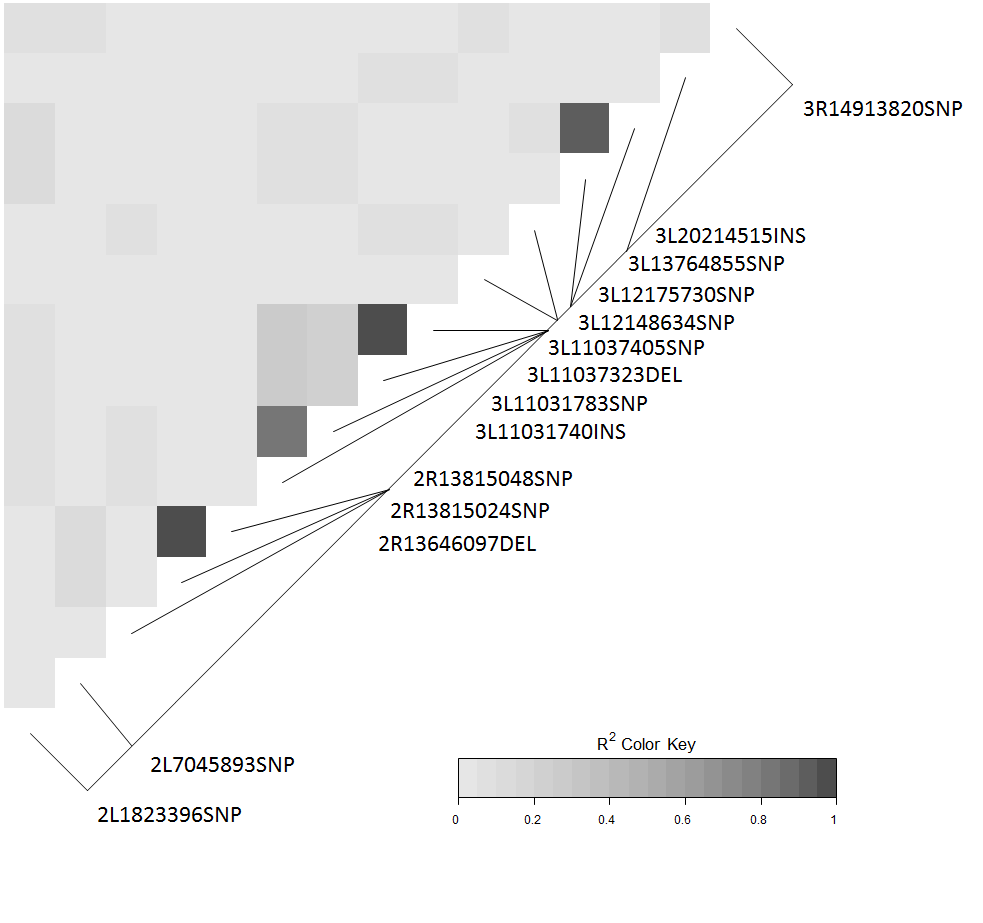

Supplement: S5 Fig — (TIF) [file pone.0132846.s005.tif]
